# Density of bulk trap states of hybrid lead halide perovskite single crystals: temperature modulated space-charge-limited-currents

**DOI:** 10.1038/s41598-019-40139-y

**Published:** 2019-03-04

**Authors:** Jan Pospisil, Oldrich Zmeskal, Stanislav Nespurek, Jozef Krajcovic, Martin Weiter, Alexander Kovalenko

**Affiliations:** 1 Brno University of Technology, Faculty of Chemistry, Materials Research Centre, Purkyňova 118, 612 00, Brno, Czech Republic; 2 University of West Bohemia, Faculty of Electrical Engineering, Plzen, 306 14 Czech Republic

## Abstract

Temperature-modulated space-charge-limited-current spectroscopy (TMSCLC) is applied to quantitatively evaluate the density of trap states in the band-gap with high energy resolution of semiconducting hybrid lead halide perovskite single crystals. Interestingly multicomponent deep trap states were observed in the pure perovskite crystals, which assumingly caused by the formation of nanodomains due to the presence of the mobile species in the perovskites.

## Introduction

Organometallic and inorganic halide perovskites are prospective candidates to replace conventional inorganic materials not only in the photovoltaic application^1–8^ but also, in solid states lasers^9^, light-emitting diodes^10^, photodetectors^11^ and solar fuels production^12^. Lead halide perovskites possess high-absorption coefficients, long-ranged ambipolar transport^13^ and low cost and facile deposition techniques such as coating and printing^14–16^. Moreover, it has been reported on facile growth of large monocrystals of lead halide^17–19^ (or even lead free^20^) perovskites by various techniques. Such single crystals, possessing very low defect density, are good candidates to disclose a variety of interesting and important properties of this group of materials. Perovskite single crystals have shown interesting properties, e.g., perovskite solar cells can harvest below the bandgap^21^, and perovskite photodetectors possess impressive characteristics for the detection of various wavelengths^22^. Furthermore, single crystals of lead halide perovskites are high-gain materials for lasing because of their high absorption coefficient, high photoluminescence quantum yield, slow Auger recombination rate, long carrier diffusion length and low defect density^23,24^.

Alongside with the other interesting properties of the lead halide perovskites, these materials possess two types of conductivity: electronic and ionic one^25^. Ionic conductivity in lead halide perovskite is the result of cations and/or anions migration across the perovskite under the influence of an electric field. As the result of the ionic diffusion, the open regions or the significant population of vacancies on the appropriate sublattice of perovskite lattice, which allow the ionic movement, appear. Aforesaid vacancy assisted ionic defects act as traps for charge carriers in the perovskite. Therefore the ionic diffusion in lead halide perovskites results in the appearance of lattice defects, which has important implications in terms of long-term stability and performance of perovskite-based devices (i.e. solar cells, LEDs, photodetectors etc.). Moreover polarization of the solar cell electrodes is usually associated with mobile ions and surface carrier recombination, and achievable open-circuit voltage^26,27^. In this regard, the understanding of the complex charge carrier dynamics induced by the ion migration is highly important. However it has to be noted, that kinetics of the mobile ions in the perovskites is a complex multicomponent phenomenon, which is still poorly understood. The existence of several ionic species, which can be a subject to diffusion, make the experimental evaluation rather obscure. As an example, it has been predicted theoretically and measured experimentally^28^, that, in fact three types of ionic species can be associated with vacancy-assisted ionic conductivity. The activation energy values to provoke the ionic movement were evaluated as: 0.58, 0.84 and 2.31 eV for I^−^, MA^+^ and Pb^2+^ respectively.

In the present communication the MAPbBr_3_ perovskite single crystals were studied by temperature-modulated space-charge-limited current (TMSCLC) method^29–32^. While regular SCLC technique is based on the measurement of current-voltage characteristic (steady-state regime) or time-of-flight of charge carriers (dynamic regime) to get information concerning the current non-linearity, charge carrier concentration, hole or electron mobility, and charge trapping process in various device architectures and materials, the TMSCLC technique is suggested as a self-consistent spectroscopic method for the determination of both the distribution of localized states (traps) and their energy. The spectroscopic character of the method follows from the simultaneous measurement of space-charge current on both voltage and temperature (energy window associated with the Fermi-Dirac statistics and the shift of the Fermi level).

## Results and Discussion

**Figure 1:**
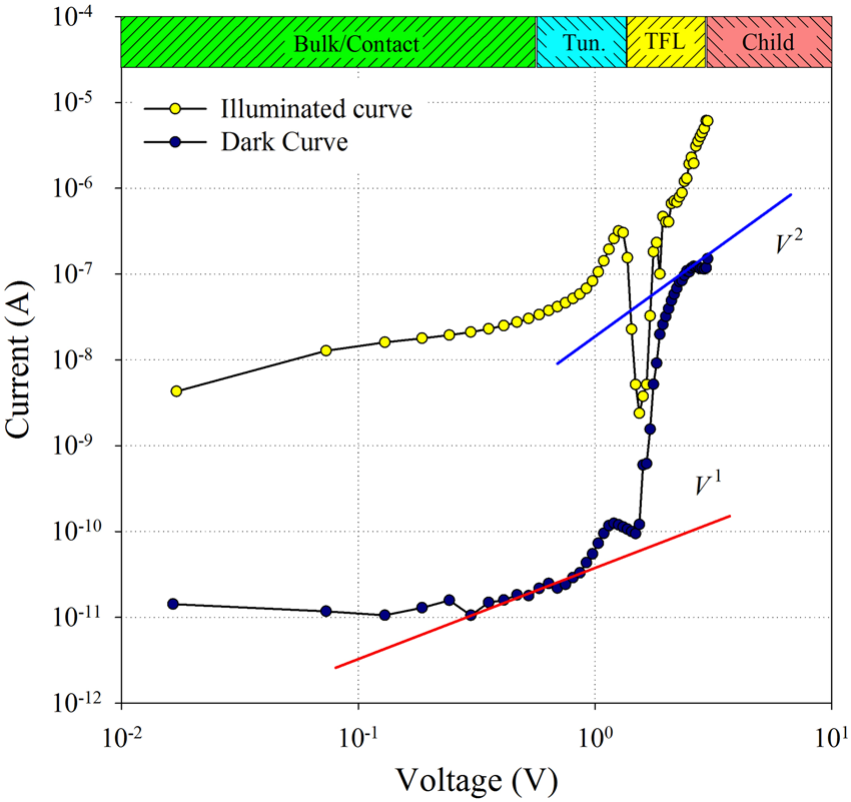
Dark *I-V* characteristic (dark blue points) and *I-V* characteristic under illumination (yellow points).

As it can be seen from the experimental current-voltage characteristic (Fig. 1), the current is influenced by the barrier up to 0.3 V, it is ohmic (*I*~*V*) in the voltage range (0.3–1.2) V. Then it is a superlinear dependence (*I*∼*V*^*m*^) with non-constant exponent *m* (*m* increases with voltage). It suggests the presence of charge carrier traps distributed in energy. The current decrease was observed in the voltage range (1.5–1.8) V. For higher voltages the *I*–*V* characteristic is typical for the material with Gaussian distribution of traps for charge carriers^33,34^. The last part of *I*–*V* characteristic (voltages higher than about 2 V) can be expressed by Child’s law (trap-free SCLC conduction). Here, for the current density *j* we can write

1 $$j=\frac{9}{8}\mu \,{\varepsilon }_{0}{\varepsilon }_{{\rm{r}}}\frac{{V}^{2}}{{L}^{3}}$$

where *μ* is the charge carrier mobility, *ε*_0_ is the permittivity of vacuum, *ε*_r_ is the relative permittivity, *V* is the voltage, and *L* is the sample thickness. From this equation the trap free charge carrier mobility can be determined for *ε*_r_ = 25.5^35^ as *μ* = 17.8 cm^2^V^−1^s^−1^.

The open question is the decrease of current in the voltage region (1.5–1.8) V. Here, we assume that the “negative differential resistance” is associated with the crystal polarization. As it was reported previously, lead halide perovskites can exhibit unipolar self-doping properties. Thus, depending on the composition of intrinsic point defects the material can be either n- or p-type^36,37^. Hence, due to the presence of the mobile ionic species in lead halide perovskites, biasing the sample results in the crystal polarization, which in its turn may be considered as an appearance of the depletion region between highly n-doped or n^++^ (i.e. MA^+^ rich or Br^−^ poor) and highly p-doped or p^++^ polar nanodomains^38^. The increasing voltage at the “N-shaped” *I*-*V* curve is presumably caused by the majority charge carriers tunneling through the depletion region between the MA^+^ and Br^−^ ion rich states at the bias voltage (1.2–1.8) V for the MAPbBr_3_. At higher voltage the charge carriers injected across the depletion region can be observed.

It follows from equations below that each point in the SCL current-voltage characteristic comprises the information about the trap density which influences the current value at the given Fermi level *E*_F_. The expression for the current density *j* in the SCLC regime can be written as:

2 $$j=\mu \,{\varepsilon }_{0}{\varepsilon }_{{\rm{r}}}\Theta \frac{(1-\gamma )\,{(2-\gamma )}^{2}{V}^{{\rm{2}}}}{{L}^{3}},$$

where $\gamma ={\rm{d}}\,\mathrm{ln}\,V/{\rm{d}}\,\mathrm{ln}\,j$ is the slope of logarithmic dependence of *I-V* characteristics, and $\Theta ={n}_{{\rm{f}}}/{n}_{{\rm{t}}}$ is the ratio of the free (*n*_f_) and trapped (*n*_t_) charge carrier concentration.

The concentration of trapped charge carriers at the Fermi level (*E*_F_) is equal

3 $${n}_{{\rm{t}}}=\frac{{\varepsilon }_{0}{\varepsilon }_{{\rm{r}}}(1-\gamma )\,(2-\gamma )V}{e{L}^{2}}.$$

Traps below the Fermi level are usually all occupied and the relation is valid

4 $${n}_{{\rm{t}}}(E)\approx {N}_{{\rm{t}}}(E)H(E-{E}_{{\rm{F}}}),$$

where *E* is the trap energy, *N*_t_ is the concentration of electronic states, and *H*(*E* − *E*_F_) is the Heaviside step function. Thus, using the slope of the current-voltage characteristic $\gamma ={\rm{d}}\,\mathrm{ln}\,V/{\rm{d}}\,\mathrm{ln}\,j$, we can determine the density of the localized electronic states.

Using the equation $j=\sigma F=e\mu {n}_{{\rm{f}}}(2-\gamma )\,V/L$, where $\sigma =e\mu {n}_{{\rm{f}}}$ is electric conductivity and $F=(2-\gamma )\,V/L$ is electric field, it is possible to determine the position of the Fermi level with the relation to the band energy levels (i.e. valence or conduction bands)

5 $${E}_{{\rm{V}}}-{E}_{{\rm{F}}}={k}_{{\rm{B}}}T[\mathrm{ln}(j/V)-\,\mathrm{ln}(2-\gamma )-\,\mathrm{ln}(e\mu {N}_{V}/L)],$$

where *E*_V_ is energy of valence band and *N*_V_ is its states concentration. In ohmic region (γ = 1) is this position proportional to activation energy ${E}_{{\rm{a0}}}={E}_{{\rm{F0}}}-{E}_{{\rm{V}}}$, where *E*_F0_ is position of Fermi level in thermodynamic equilibrium.

**Figure 2:**
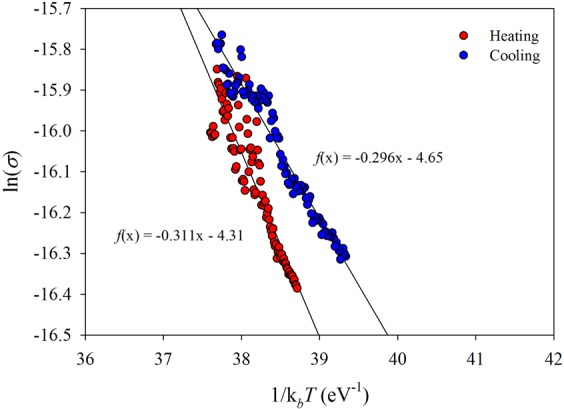
Activation energy of the SCLC current at heating (red scatter plot) and cooling (blue scatter plot) at applied voltage is 2.35 V. The slope of the linear equation represents the activation energy.

For this reason the activation energy of the current (conductance) was measured for each applied voltage in range of (0–3) V, with step 28 mV. The temperature was changed (modulated) in the range of (0–40) °C. Figure 2 shows the level of accuracy at the measurement of activation energy according to the relation $\sigma =(I/V)(L/S)={\sigma }_{0}\exp (-{E}_{{\rm{a}}}/{k}_{{\rm{B}}}T)$ for heating and cooling cycle. Here, *L* is the distance between the electrodes of the certain area *S*. The slope of these dependences is directly activation energy.

An approximate relation can be considered: ${E}_{{\rm{a}}}\approx {E}_{{\rm{F}}}-{E}_{{\rm{V}}}$ and $\Delta {E}_{{\rm{a}}}\approx \Delta {E}_{{\rm{F}}}$. The shift of the Fermi level Δ*E*_F_ can be determined according to the relation mentioned above. Note, that Fermi level position represents the energy at which the localized state (trap) is filled during the measurement of current-voltage characteristic.

**Figure 3:**
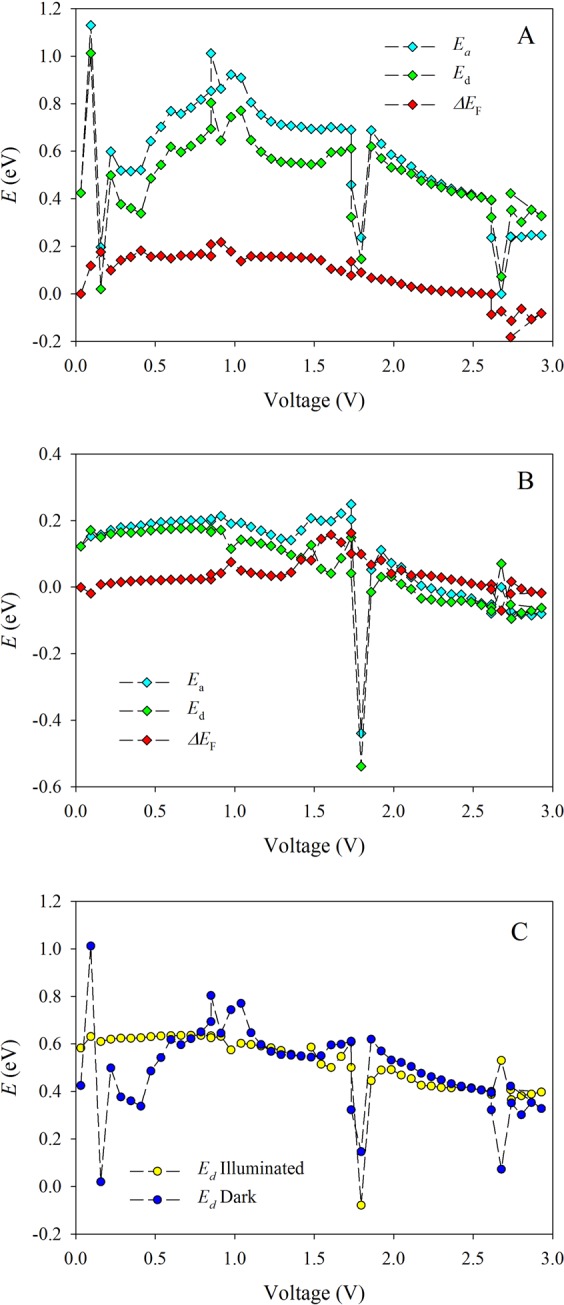
Dependence of activation energy *E*_a_, Fermi level shifts Δ*E*_F_ and dominant energy *E*_d_ = *E*_a_ − Δ*E*_F_ on the applied voltage^32^ in the dark (**A**), under illumination (**B**) and dependence of both dominant energies (dark/illumination) on the voltage. Note that under light illumination the dominant energy is shifted for 0.46 eV, see the text for details.

Figure 3 represents the activation energy and Fermi level shift for the dark (A) and illuminated (B) sample (each point in the plot is averaged from 300 experimental values). Note, that for the low voltages (subohmic regime, *V* < 0.3 V) where the Schottky barrier is predominant, traps also significantly influence the activation energy. Here, the dependence of the Fermi level on the initial voltage value (*V*_0_ ≈ 0.03 V)

6 $${\rm{\Delta }}{E}_{{\rm{F}}}={k}_{{\rm{B}}}T[\mathrm{ln}\,\frac{j}{{N}_{{\rm{c}}}(2-\gamma )V}-\,\mathrm{ln}\,\frac{{j}_{0}}{{N}_{{\rm{c}}}(2-{\gamma }_{0}){V}_{0}}]$$

and the dominant energy determined as the difference between the activation energy and the shift of the Fermi level *E*_d_ = *E*_a_ − Δ*E*_F_^32^, both in the dark and under the illumination. From the Fig. 3C in which the two dominant energies are plotted together, it can be observed that the dominant energy under illumination being shifted by 0.46 eV to higher values, which indicates that the charge trapping takes place in the same states

From the Fig. 3 follows that three main trap states (*E*_t_) influence the electric behavior of the sample under study *E*_t_ = 0.63 eV, 0.55 eV, and 0.38 eV. The activation energy *E*_a_ = 0.80 eV is associated with the thermodynamic equilibrium position of the Fermi level. During the charge injection (the superlinear *I*–*V* characteristic), the charge carriers is influenced by traps. The deepest trap *E*_t_ = 0.63 eV, can be related to the vacancy assisted mobile ionic species (presumably MA^+^); thus in the range of (1.1–1.4) V of the current-voltage characteristic we observe trap filled limit for p-type charge carriers. At higher voltages the interchange of dominant charge carriers is observed, which is represented by the significant perturbations in activation energy. In this region a negative differential current is observed. The interchange between the dominant charge carriers (MA^+^ vs. Br^−^), can be observed as a tunneling through the depletion region between p^++^ and n^++^ sites.

Under the illumination, the Fermi level is strongly influenced by the light generated charge carriers (electrons), From the overall thermal conductivity measurement the Drude-like behavior is expected^39^. The dominant energy is significantly lower (0.17 V); no contact barrier was observed (Fig. 3B). The dominant states close to the valence band (0.09 eV) are taken over by the charge carrier traps above the edge of the valence band (−0.08 eV). As a result (see Fig. 3C), three trap states with the energies *E*_t_ = 0.17 eV, 0.09 eV, and −0.08 eV are formed under illumination. These are the same relationships as in the dark, but they are related to the other position of the edge of the valence band (shifted by 0.46 eV to lower values).

Under the assumption, that the concentration of states in the valence band of the MAPbBr_3_ crystal equals to ${N}_{{\rm{V}}}\approx 6\times {10}^{17}{{\rm{cm}}}^{-3}$ and crystal thickness is $L\approx {10}^{-3}{\rm{m}}$, the mobility for holes was determined in the dark conditions as ${\mu }_{{\rm{p}}}=17.8$ cm^2^V^−1^s^−1^.

**Figure 4:**
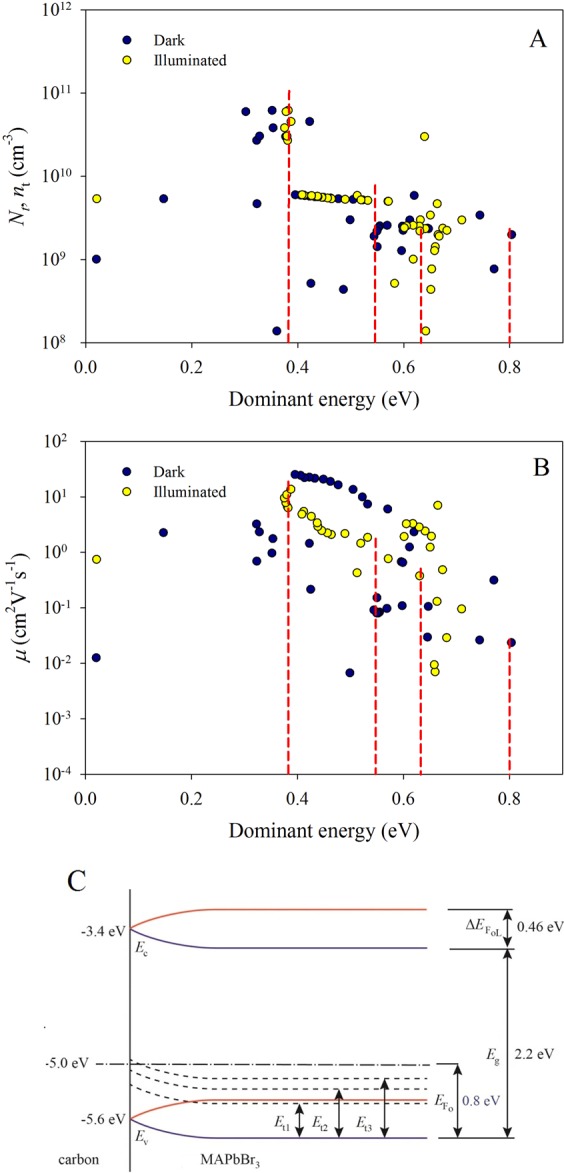
Dependences of concentration of the charge carriers (**A**) and drift mobility versus the dominant energy. Concentration of states and mobility at dominant energies (**B**) of the traps are depicted as dashed (see Table 1). Band gap diagram of carbon/MAPbBr_3_ (**C**) Schottky barrier blue and red lines for the dark and illuminated conditions respectively, dominant energies of the traps depicted as dashed.

Using the expression for trapped charge carriers (equation 3) it is possible to calculate their concentration for various energies and therefore to get energy distribution of traps. For the more representative dependence of the charge carriers on individual traps the charge carrier concentration vs. activation energy $g(E)\approx {\rm{d}}{n}_{{\rm{tL}}}/{\rm{d}}{E}_{{\rm{F}}}$ (Fig. 4A) can be plotted. The red dashed vertical lines represent individual charge carrier traps as it was estimated from the *I-V* characteristics. It can be clearly seen how the concentration of the charge carrier increases at each individual trap states, which means charge carriers accumulation at the trap density. For the above-mentioned calculation the relative permittivity of the MAPbBr_3_ was taken as ${\varepsilon }_{{\rm{r}}}\approx 25.5$^35^. Generally, the values for the concentrations and charge carrier mobilities obtained by the TMSCLC method are close to the values reported before^40–44^. In the Fig. 4B the concentrations were calculated using the equation (2).

Interestingly, the band diagram (see Fig. 4C) shows, that the charge transfer is associated only with holes in the valence band. Trap state *E*_t1_ is located above the edge of the valence band, when no light illumination is applied, however, under light illumination it shifts under the edge of the valence band.

The results for all the determined traps are summarized In the Table 1: their position (dominant energy is equal to activation energy when the trap is fulfilled ${E}_{{\rm{d}}}\approx {E}_{{\rm{a}}}$), the concentration of trap states for this case (${N}_{{\rm{t}}}\approx {n}_{{\rm{t}}}$) and drift mobility of the charge carriers in the traps (${\mu }_{{\rm{d}}}\approx \Theta {\mu }_{{\rm{0}}}$, where *μ*_0_ is microscopic mobility), it can be determined from Childs law (when $\Theta =1$, see Eq. 1), or from Fermi level position in ohmic regime (${E}_{{\rm{F0}}}\approx {E}_{{\rm{a}}}$, when ${E}_{{\rm{V}}}=0\,{\rm{eV}}$, see Eq. 5)

**Table 1:** Dominant energies, concentrations of trap states, and drift mobilities of charge carriers MAPbBr_3_ *E*_F0_ = 0.8 eV).

Dark	Light	Dark/Light	
*E*_t_ (eV)	*E*_t_ (eV)	*N*_t_ (cm^−3^)	*μ* (cm^2^V^−1^s^−1^)
0.63	0.17	2.4 × 10^9^	1.6
0.55	0.09	4.5 × 10^9^	9.1
0.38	−0.08	6.2 × 10^10^	17.8

Comparing current results with previously reported, it has to be emphasized, that the mobility values in lead halide perovskite may vary within the several orders of magnitude^43,45^, whilst theoretically, mobilities in lead halide perovskite supposed to be comparable with the ones of inorganic semiconductors, e.g. GaAs, as far as it has only slightly lower effective masses for conduction band electrons and valence band holes, however there are some limiting factors for the charge carrier mobilities in perovskites, which are extrinsic and intrinsic effects.

**Figure 5:**
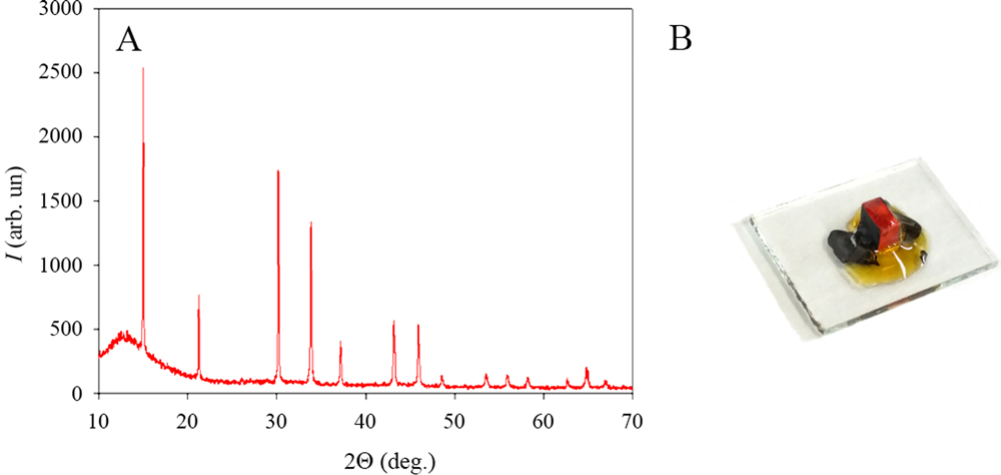
XRD pattern of the single crystal MAPbBr_3_ perovskites (**A**). Graph inset: single crystal device prepared with the patterned ITO covered glass and contacted with carbon paste.

Intrinsic effects cannot be avoided, as far as they are originated result from charge-carrier interactions with the crystal lattice. On the other hand extrinsic effects are the result of material imperfections, such as grain boundaries, energetic disorder, or impurities. In the present case, the growth of the crystals was precisely controlled, thus regular rectangular shaped samples were obtained (see Fig. 5B), Moreover, it is assumed, that the carbon contacts prepared from the non-polar solvent xylene (which is antisolvent for the perovskite^46^) resulted in contact interface with less defects, in comparison with the samples where thermally evaporated contacts were deposited.

## Experimental

As it is shown in Fiugure 6, macroscopic 2–5 mm sized (MAPbBr_3_) perovskite single crystals were prepared from a solution by an inverse temperature crystallization method without a nucleation^35^. Lead bromide (PbBr_2_, 99.999%, Sigma-Aldrich), methylammonium bromide (CH_3_NH_2_.HBr, 98%, Sigma-Aldrich) and dimethylformamide (DMF, 99.8%, Sigma-Aldrich), were used as received without further purification. 1 M solution of PbBr_2_ and CH_3_NH_2_.HBr in DMF was added to ultrasonic bath under Ar atmosphere at room temperature for 3 min. The transparent solution was filtered using PVDF filter (pore size 0.45 μm). The resulting filtrate was gradually heated up in the oil bath from the room temperature up to 80 °C for about an hour, and consequently temperature was kept constant to obtain the crystals of the desired size. Then obtained crystals were rinsed in diethylether, dried with argon gun and instantly transferred to the glove box with a nitrogen atmosphere. Contacts were deposited at two opposite facets with a non-polar solvent (hexane) carbon paste, with the subsequent encapsulation with Ossila epoxy resin to avoid any influence of the atmospheric oxygen and moisture, when the electrical measurements outside of the glove box were performed.

XRD spectra (MAPbBr_3_) showed diffraction angles typical for perovskite. Moreover, in our previous work^18^ we have measured 2D XRD mapping of the samples prepared by the same method, which proved the single crystal nature of the samples.

Current- or photocurrent-voltage characteristics for TMSCLC method were measured using Keithley 2410 Source Meter in the interval (0–3) V with 0.028 V step. At each step the temperature was changed from 0 to 40 °C, using Lauda ECO Silver RE 415 thermostat and measured with thermocouple of type K by Agilent 34420 A, Digit NanoVolt/MicroOhm Meter. The current-voltage response was measured for 3 minutes at each point, in this regards the measurements are considered as steady-state.

For photoconductivity measurements the sample was irradiated by the white LED lamp with the intensity of 34 W/m^2^.

## Conclusion

To sum up, in the present paper the implementation of the temperature-modulated space-charge-limited-current spectroscopy is applied to the single crystal methylammonium lead bromide perovskites. As a result, charge carrier mobilities (holes in the valence band) were calculated as 1.6, 9.1 and 17.8 cm^2^V^−1^s^−1^. Furthermore, with no illumination applied the activation energy *E*_a_ = 0.80 eV is associated with the thermodynamic equilibrium position of the Fermi level, additionally three individual trap states were found with the activation energies 0.63, 0.55 and 0.38 eV and concentrations of 2.4 × 10^9^, 4.5 × 10^9^, 6.2 × 10^10^, cm^−3^ respectively, notably under the light illumination *E*_t_ = 0.38 shifts under the edge of the valence band resulting in Drude-like model.
